# A Phenolphthalein-Dummy Template Molecularly Imprinted Polymer for Highly Selective Extraction and Clean-Up of Bisphenol A in Complex Biological, Environmental and Food Samples

**DOI:** 10.3390/polym10101150

**Published:** 2018-10-15

**Authors:** Jiajia Yang, Yun Li, Chaonan Huang, Yanna Jiao, Jiping Chen

**Affiliations:** 1 College of Materials Science and Engineering, Hebei University of Engineering, 199 South Guangming Street, Handan 056038, China; yjj177@163.com; 2 CAS Key Laboratory of Separation Sciences for Analytical Chemistry, Dalian Institute of Chemical Physics, Chinese Academy of Sciences, 457 Zhongshan Road, Dalian 116023, China; liyun@dicp.ac.cn (Y.L.); huangchaonan@dicp.ac.cn (C.H.); 3 University of Chinese Academy of Sciences, Beijing 100049, China; 4 Inspection and Quarantine Technology Centre, Hunan Entry-Exit Inspection and Quarantine Bureau, Changsha 410004, China; jiaoyn2013@163.com

**Keywords:** molecularly imprinted polymer, dummy template, solid-phase extraction, bisphenol A, biological, environmental and food samples

## Abstract

A molecularly imprinted polymer (MIP) for highly selective solid-phase extraction (SPE) of bisphenol A (BPA) was prepared using phenolphthalein (PP) as the novel dummy template by bulk polymerization. A particle diameter distribution of 40–60 μm, a specific surface area of 359.8 m^2^·g^−1^, and a total pore volume of 0.730 cm^3^·g^−1^ for the prepared PP-imprinted polymer (PPMIP) were obtained. Good selectivity and specific adsorption capacity for BPA of the prepared PPMIP were also demonstrated by the chromatographic evaluation and sorption experiments. The PPMIP as a SPE sorbent was evaluated for the selective extraction and clean-up of BPA from complex biological, environmental, and food samples. Meanwhile, an accurate and sensitive analytical method based on the PPMIP-SPE purification procedure coupled with high performance liquid chromatography-diode array detector (HPLC-DAD) detection has been successfully developed for the rapid determination of BPA from these samples, with detection limits of 1.3 ng·mL^−1^ for bovine serum and milk, 2.6 ng·mL^−1^ for human urine and edible oil, 5.2 ng·mL^−1^ for soybean sauce, and 1.3 ng·g^−1^ for sediment. The BPA recoveries at two different spiking levels were in the range of 82.1–106.9%, with relative standard deviation (RSD) values below 7.7%.

## 1. Introduction

Bisphenol A (BPA, as shown in Figure S1) is an industrial chemical used in numerous products such as polycarbonate plastics and epoxy resins, food and beverage packing, dental sealants, and thermal paper [1], and its adverse effects on reproduction and development, neural network, cardiovascular, metabolic, and immune systems in humans have been well demonstrated [2,3]. In recent years, BPA has been widely found in a variety of biological samples [4,5], environmental matrixes [6,7] and food products [8,9]. Hence, investigations on the occurrence of BPA in biological, environmental, and food samples, which are essential for human exposure assessments, have drawn great attention worldwide [10,11,12]. Nevertheless, the low concentrations of BPA and the complex nature of these samples become bottlenecks for the accurate determination of BPA. In this regard, there has been a growing interest in developing commercial or laboratory-made sorbents with high selectivity and sensitivity towards BPA [13,14].

Molecular imprinting is known as a technique for generating tailor-made recognition sites with memory of the shape, size, and functional groups of the template molecules [15], and it has been widely used in the application of chemical separation [16]. The utilization of the molecular imprinting technique in solid-phase extraction (SPE), so-called molecularly imprinted solid-phase extraction (MISPE), has become one of the most popular strategies for the pretreatment of complex biological, environmental, and food samples [17,18,19]. Currently, a lot of research work on MISPE is still based on organic polymers, due to the merits of good reusability and chemical stability [20,21]. Meanwhile, bulk polymerization is considered as the most popular and general method for the preparation of molecularly imprinted polymers (MIPs), due to its rapidity and simplicity [22,23]. When MIPs are prepared using the target compound as template, the leakage of trace amounts of the template molecules has been considered to be damaging to the accurate quantification of analytes during the procedure of trace level analysis [24]. Hence, when MIP is used for trace analysis, it is not advisable to use the target molecule as the template. This problem can be solved by using a dummy template instead of the target compound, since any leakage will be different from the analytes, such as the most commonly used isotope labeled templates [25,26] and structural analogs of analytes [27,28]. Despite possessing a high specificity when used as a dummy template, isotope-labeled compounds suffer the disadvantages of high cost and sometimes commercial unavailability. Meanwhile, the use of mass spectrometry for detection and the potential isotopic contamination also limit their wide applications in real analysis [27]. Consequently, for the target analyte BPA, much effort has been expended in the use of related structural analogs, such as bisphenol S (BPS) [28], bisphenol B (BPB) [29], and bisphenol AF (BPAF) [30]. However, these BPA structural analogs still belong to the same kind of hazardous pollutants [2], which are not favorable for green chemistry. Therefore, for BPA analysis, searching for more appropriate and efficient dummy templates would be highly desirable.

Phenolphthalein (PP, Figure S1) is a weak organic acid, and it is often used as an indicator in acid-based titrations. In our preliminary work, PP was found to be structurally close to BPA, and the developed PP imprinted polymer (PPMIP) had good selectivity towards BPA [31]. As a result, PP can be considered as an ideal dummy template candidate for BPA, due to its relatively lower toxicity, cheap price and easy availability. However, the developed PPMIP had not been further explored for BPA analysis in a real sample.

This study aims to fabricate the PPMIP material by bulk polymerization and apply it for highly selective extraction of BPA from two biological samples (human urine and bovine serum), one environmental sample (sediment), and three food samples (milk, edible oil and soybean sauce). In the present work, BPA imprinted polymer (BPAMIP) coupled with BPA analogues, including bisphenol B (BPB), bisphenol AF (BPAF), bisphenol S (BPS), 17*β*-estradiol (E2), diethylstilbestrol (DES), and 4-nonylphenol (NP) (as shown in Figure S1) were used to evaluate the cross-selectivity of the prepared PPMIP. Finally, the PPMIP-SPE coupled with the high performance liquid chromatography-diode array detector (HPLC-DAD) method was successfully developed for highly selective extraction and rapid detection of BPA in complex human urine, bovine serum, sediment, milk, edible oil, and soybean sauce samples. Compared with other previous studies [17,18,28,29,30], a higher imprinting factor (*IF*) value for BPA was obtained, and more real samples were tested in this work.

## 2. Materials and Methods

### 2.1. Chemicals and Materials

Ethylene glycol dimethacrylate (EGDMA, 99%), 4-vinylpyridine (4-VP), HPLC-grade trifluoroacetic acid (TFA), 2,2′-azobisisobutyronitrile (AIBN, 99%), BPAF (99%) and BPS (>98%) were purchased from J&K Scientific (Beijing, China). BPA (>99%), BPB (>98%) and E2 (>97%) were obtained from Tokyo Chemical Industry Co. (Tokyo, Japan). DES (99%) and NP (99.3%) were purchased from Dr. Ehrenstorfer GmbH (Augsburg, Germany). Sodium hydroxide was purchased from Tianjin Damao Chemical Reagents Factory (Tianjin, China). HPLC-grade methanol and acetonitrile (ACN) were supplied by Fisher Scientific (Fair Lawn, NJ, USA). Dichloromethane (DCM) was provided by J.T. Baker (Philipsburg, NJ, USA). Hydrochloric acid and acetone were obtained from Beijing Chemical Works (Beijing, China). Acetic acid was purchased from Hengxing Chemical Preparation Co. (Tianjin, China). All solvents were analytical grade unless noted otherwise. The water used in all experiments was deionized water purified by a Milli-Q system (Millipore, Billerica, USA). The empty SPE cartridges (polypropylene, 3 mL) with frits (polyethylene) were purchased from Sipore (Dalian, China).

### 2.2. Preparation of the PPMIP, BPAMIP, and Non-Imprinted Polymer (NIP)

The PPMIP material was made as follows: 1 mmol (0.318 g) of PP, 4 mmol (0.42 mL) of purified 4-VP, and 20 mmol (3.8 mL) of purified EGDMA, coupled with 40 mg of AIBN were pre-dissolved in 5.6 mL of ACN (porogen solvent) within a glass tube (20 mL). The obtained solution was saturated with high purity nitrogen for 15 min to remove the dissolved oxygen, and then kept at 4 °C for 2 h under a nitrogen atmosphere. Afterwards, the glass tube was sealed and placed in a water bath at 60 °C for 24 hr. The obtained white block of PPMIP material was crushed, ground, and sieved, and polymer particles in the size range of 40–60 μm were collected. Then, the polymer particles were precipitated in acetone to remove the adsorbed fine particles, and the resulting PPMIP particles were dried overnight under a vacuum. Finally, the PP template was extracted from the PPMIP polymer using Soxhlet extraction by methanol/acetic acid (9:1, *v*/*v*) for 24 h. BPAMIP and NIP were prepared simultaneously using 1 mmol (0.228 g) of BPA as template molecules and in the absence of the template molecules by the same protocol, respectively.

### 2.3. Characterization of the PPMIP, BPAMIP, and NIP

Nitrogen adsorption–desorption isotherms were measured using a NOVA 4000 surface area analyzer (Quantachrome, Boynton Beach, FL, USA). Before measurements, the obtained PPMIP, BPAMIP, and NIP particles were outgassed at 100 °C for 8 h. The specific surface areas and pore volumes of the PPMIP, BPAMIP, and NIP particles were calculated using the standard Brunauer-Emmett-Teller (BET) method, and their pore size distributions were calculated using Barrett-Joyner-Halenda (BJH) theory. Scanning electron microscopy (SEM) images were obtained via a JEOL JSM-7800F microscope (Tokyo, Japan).

### 2.4. Chromatographic Evaluation Experiments

The PPMIP, BPAMIP, and NIP particles were slurry-packed into three HPLC columns (100 mm × 4.6 mm i.d.) using a HY-HPLC-S packing pump (Hydrosys Industries, Beijing, China) under 3000 psi with methanol as solvent, and the obtained HPLC columns were named as PP-imprinted, BPA-imprinted, and non-imprinted columns, according to the type of their packing materials. Chromatographic analyses were performed with an HPLC system equipped with a Rheodyne manual injector, a Waters 515 pump, and a Waters 2487 UV detector.

The chromatographic evaluation was performed by injecting 20 μL of each analyte (20 ppm) with a mobile phase (ACN) flow rate of 1 mL·min^−1^, and 220 nm was selected as the detection wavelength. The capacity factor (*k*) was calculated according to Equation (1):

*k* = (*t*_R_ − *t*_0_)/*t*_0_ (1)

where *t*_R_ and *t*_0_ are the retention times of the analyte and the void marker (acetone), respectively. The *IF*s of PPMIP for BPA, BPB, BPAF, BPS, E2, DES, and NP were calculated by Equation (2), and the *IF*s of BPAMIP for these analytes were calculated by Equation (3):

*IF* = *k*_PPMIP_/*k*_NIP_ (2)

*IF* = *k*_BPAMIP_/*k*_NIP_ (3)

where *k*_PPMIP_, *k*_BPAMIP_ and *k*_NIP_ are the capacity factors of the analytes on the PP-imprinted, BPA-imprinted, and non-imprinted columns, respectively.

### 2.5. Sorption Experiments

PPMIP and NIP particles (40.0 mg for each material) were separately mixed with 2.0 mL of BPA solutions with various concentrations (0.02–4.0 mmol·L^−1^) in ACN. The mixtures were shaken at 150 rpm at 25 °C for 24 h, and then they were filtered through a membrane (0.22 μm). The free concentrations of BPA in the filtrates were determined by the developed HPLC method. The adsorption capacity and the equilibrium dissociation constant (*K*_d_, mmol·L^−1^) of PPMIP and NIP were calculated according to Equations (4) and (5) [17]:

*Q* = (*C*_0_ − *C*_f_)*v*/m (4)

*Q*/*C*_f_ = −(1/*K*_d_)*Q* + *Q*_max_/*K*_d_ (5)

*C*_0_ (μmol·L^−1^) and *C*_f_ (μmol·L^−1^) are the initial and final concentrations of BPA, respectively; *v* (L) is the sample total volume, m (g) is the mass of the used polymer material; *Q* and *Q*_max_ (μmol·g^−1^) are the BPA adsorption amounts at equilibrium and saturation, respectively.

### 2.6. Analytical Procedures

#### 2.6.1. Real Sample Preparation

Human urine samples were collected from three volunteers (male). The obtained samples were centrifuged at 6000× *g* for 15 min to remove precipitates. A total of 1 mL of human urine spiked with BPA (100 and 500 ng) was diluted to 10 mL with water (pH was adjusted to 9.0). Bovine serum samples were obtained from Sijiqing Co. (Hangzhou, China). A total of 2 mL of bovine serum spiked with BPA (100 and 500 ng) was deproteinated by the addition of ACN (4 mL). After vortexing for 1 min, the obtained sample was centrifuged at 3000× *g* for 5 min at 4 °C. Then, the supernatant was transferred into a glass tube. The extraction step was repeated twice, and the combined extracts were concentrated to about 3 mL under nitrogen gas. At last, the concentrated extracts were diluted to 10 mL with water.

Sediment samples were collected from Liaohe River at three locations (Liaoning, China). Then, the obtained samples were freeze-dried, ground, and passed through a sieve (2 mm). The extraction of sediment samples was based on the procedure reported by Sun et al. [18]. In brief, 2 g of sediment spiked with BPA (100 and 500 ng) was extracted with methanol (5 mL) by shaking for 60 min. After centrifugation at 4500× *g* for 5 min, the supernatant was transferred into a glass tube. The extraction step was repeated twice, and the combined extracts were concentrated to 1 mL under nitrogen gas. The concentrated extracts were then diluted to 10 mL with water.

Milk, edible oil, and soybean sauce samples were purchased from local supermarket (Dalian, China). The preparation procedure of milk sample (2 mL) is the same as bovine serum. A total of 1 mL of edible oil spiked with BPA (100 and 500 ng) was mixed with 1 mL of DCM and 3 mL of sodium hydroxide water solution (1 mol·L^−1^). After vortexing for 10 min, the sample was centrifuged at 4000× *g* for 10 min. Then, the water phase was transferred into a glass tube. The extraction step was repeated twice, and the extracts were combined and diluted to 10 mL with water (pH was adjusted below 9.0). A total of 0.5 mL of soybean sauce spiked with BPA (100 and 500 ng) was diluted to 10 mL with water (pH was adjusted to 9.0).

#### 2.6.2. SPE Procedure

The PPMIP particles (200 mg) were packed into a SPE cartridge (3 mL) using an upper frit and a lower frit to prevent sorbent loss. The obtained cartridges were connected to a SPE manifold (Supelco Visiprep, Bellefonte, PA, USA) equipped with a vacuum pump (Jinteng, Tianjin, China). Prior to sample loading, the SPE cartridges were equilibrated with ACN (3 mL) and water (3 mL). Then, the prepared real samples were percolated at a flow rate of 1.0 mL·min^−1^. After drying under vacuum for 30 min, the SPE cartridges were rinsed with 2 mL of can, and the retained BPA were eluted with 4 mL of methanol/TFA (98:2, *v*/*v*). Finally, the eluates were evaporated to nearly dryness under nitrogen gas, and the residues were diluted to 1.0 mL with methanol/water (65:35, *v*/*v*) for further HPLC analyses.

#### 2.6.3. HPLC Analyses

An Agilent 1200 HPLC system (Santa Clara, CA, USA) equipped with an on-line degasser, an autosampler, a quaternary pump, a column incubator, and a diode array detector (DAD), was used for the determination of BPA. Chromatographic separations were carried out on a ZORBAX SB-C18 column (5 μm, 250 mm × 4.6 mm i.d., Agilent, Santa Clara, CA, USA). The mobile phase consisted of isocratic methanol/water (70:30, *v*/*v*) for the analysis of BPA in the sorption experiments. For the determination of BPA in real samples, isocratic methanol/water (65:35, *v*/*v*) was adopted. The injection volume was 5 μL for sorption experiments, and 20 μL for real sample analyses. In both cases, the column temperature was kept at 25 °C, the flow rate was 1 mL·min^−1^, and the detection wavelength was set at 225 nm.

## 3. Results and Discussion

### 3.1. Characterization of the PPMIP, BPAMIP and NIP

The porosity of the prepared PPMIP, BPAMIP, and NIP particle materials were investigated by nitrogen adsorption–desorption measurements. PPMIP, BPAMIP, and NIP all showed typical “type IV” nitrogen adsorption–desorption isotherms (Supporting Information Figure S2a–c). The BET specific surface area (*S*_BET_) and total pore volume (*V*_t_) for the PPMIP were 359.8 m^2^·g^−1^ and 0.730 cm^3^·g^−1^, respectively. The obtained *S*_BET_ and *V*_t_ for the BPAMIP were 369.6 m^2^·g^−1^ and 0.748 cm^3^·g^−1^, respectively. Meanwhile, the obtained *S*_BET_ and *V*_t_ for the NIP were 363.2 m^2^·g^−1^ and 0.709 cm^3^·g^−1^, respectively. Evidently, the *S*_BET_ and *V*_t_ for the PPMIP, BPAMIP, and NIP were very close. Furthermore, similar pore size distributions for the PPMIP, BPAMIP, and NIP were also observed, which consisted of a sharp peak at 3.3 nm for PPMIP, 3.3 nm for BPAMIP, and 3.3 nm for NIP, and a wide distribution between 2 and 50 nm without other obvious peaks (Supporting Information Figure S2a,b,c insets).

**Figure 1:**
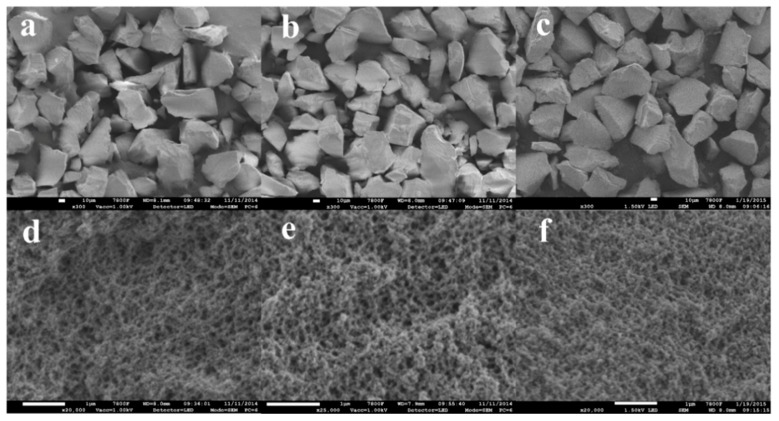
Scanning electron microscopy (SEM) images for phenolphthalein (PP)-imprinted polymer (PPMIP) (**a**, scale length: 10 μm; **d**, scale length: 1 μm), bisphenol A (BPA) imprinted polymer (BPAMIP) (**b**, scale length: 10 μm; **e**, scale length: 1 μm) and non-imprinted polymer (NIP) (**c**, scale length: 10 μm; **f**, scale length: 1 μm).

The morphologies of the prepared PPMIP, BPAMIP, and NIP particles are given by SEM microphotographs (Figure 1). As can be seen from Figure 1a–c, PPMIP, BPAMIP, and NIP were endowed with similar particle sizes (40–60 μm). As shown in Figure 1d–f, both large and small pores were observed, which were consistent with the BJH results.

### 3.2. Selectivity of the PPMIP and BPAMIP

The cross-selectivity of PPMIP and BPAMIP for six structurally related compounds (BPB, BPAF, BPS, E2, DES, and NP) were evaluated by comparing their *IF* values on both imprinted polymers, and the results are shown in Figure S3. For both PPMIP and BPAMIP columns, BPA, BPB, BPAF, and BPS showed noticeably higher *IF* values than E2, DES, and NP, mostly due to their better structural similarities towards PP and BPA. The BPAMIP column gave obviously higher *IF* values of 20.7 and 13.5 for BPA and BPB, respectively, compared to the PPMIP column (9.0 for BPA and 8.6 for BPB). Higher *IF* values for BPAF (10.6) and BPS (11.9) were obtained when the PPMIP column was adopted. Although an *IF* value of 9.0 for BPA of PPMIP was less than that of BPAMIP, it was still considered as an excellent selective recognition capability for BPA. Furthermore, compared with other previous studies, the prepared PPMIP provided a higher or comparable *IF* value for BPA (Table 1). Combining with similar characterization results between PPMIP, BPAMIP, and NIP (mainly including *S*_BET_ and pore volume), it can be concluded that the higher retention for BPA on both PPMIP and BPAMIP than NIP were mainly from the existence of imprinting sites. Therefore, by evaluating the *IF* values and physical parameters above, PP was found to be a good alternative as a dummy template for BPA.

### 3.3. Binding Properties of the PPMIP and NIP

**Figure 2:**
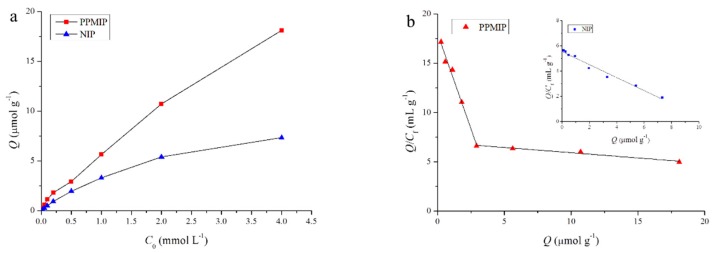
(**a**) BPA binding isotherms for PPMIP and NIP; (**b**) Scatchard plot for PPMIP (inset: Scatchard plot for NIP).

The molecular imprinting effect was further confirmed by comparing the BPA binding capacity between PPMIP and NIP. As can be seen from Figure 2a, the PPMIP displayed a noticeably higher adsorption capacity than the NIP for BPA over the tested concentration range of 0.02–4.0 mmol·L^−1^, which indicated the presence of recognition sites for BPA in PPMIP. The distinction of BPA adsorption amounts between PPMIP and NIP increased with the increase of BPA initial concentration. The obtained binding data for BPA of PPMIP and NIP were then processed with Scatchard analysis (Equation (5)), and the results were shown in Figure 2b (inset: Scatchard plot for the binding data of NIP). As can be seen from Figure 2b, two straight lines were obtained for the binding data of PPMIP. This result indicates that the binding sites in PPMIP can be classified into two different types: the specific and the non-specific binding sites. The linear regression equations for the two straight lines are Equations (6) and (7):

*Q*/*C*_f_ = 18.02 − 3.867*Q* (*r* = 0.9940) (6)

*Q*/*C*_f_ = 6.971 − 0.1062*Q* (*r* = 0.9883) (7)

where the *K*_d_ and *Q*_max_ values can be calculated from the slopes and intercepts of the two equations. The *K*_d_ and *Q*_max_ values are calculated to be 0.2586 mmol·L^−1^ and 4.661 μmol·g^−1^ for the specific binding sites (upper line), and 9.416 mmol·L^−1^ and 65.64 μmol·g^−1^ for the non-specific binding sites (lower line), respectively. Usually, lower *K*_d_ and higher *Q*_max_ values mean higher binding affinities and capacities [28]. The result of static adsorption showed that 200 mg of PPMIP material could selectively adsorb 212.8 μg of BPA.

### 3.4. Optimization of SPE Procedure

#### 3.4.1. Selection of Sample Loading pH and Volume

Different volumes (5, 10, 15 and 20 mL) of water spiked with BPA (500 ng) were adjusted to pH 3.0, 6.0, 9.0, and 12.0 by 0.1 M hydrochloric acid and sodium hydroxide. These solutions were percolated through the PPMIP-SPE cartridge at a flow rate of 1.0 mL·min^−1^. After analyses, the obtained BPA recoveries were close to 100% in the pH range of 3.0–9.0, even with a loading volume of 20 mL. Nevertheless, the breakthrough of BPA was observed at pH 12.0 due to the complete ionization of BPA under this pH condition. Owing to the very complex nature of biological fluids and food samples, a relatively small sample volume was preferably used. Finally, the sample pH range of 3.0–9.0 was chosen and a medium loading volume of 10 mL was adopted.

#### 3.4.2. Selection of Washing Solvent Volume

**Figure 3:**
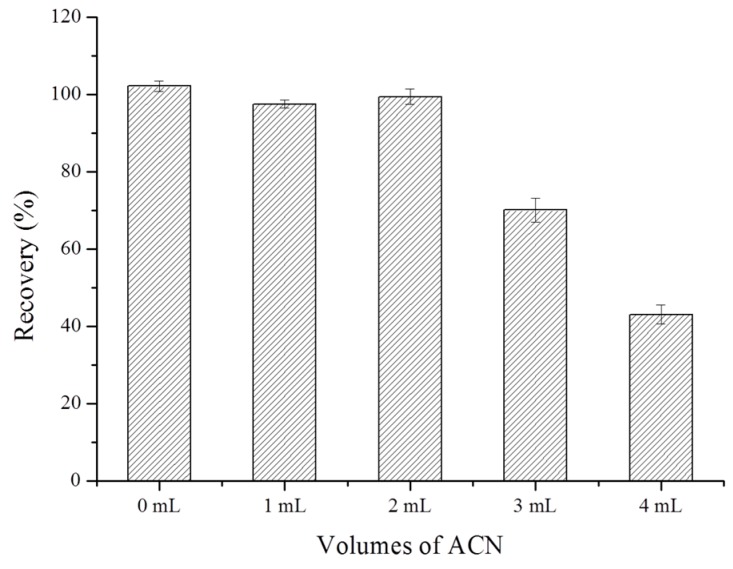
Influence of different washing solvent volumes (0, 1, 2, 3, and 4 mL) on BPA recoveries for PPMIP.

To realize the elimination of co-adsorbed matrix components in the investigated biological, environmental and food samples, an appropriate washing step using ACN as the washing solvent was adopted after sample loading. The influence of washing solvent volume on BPA recoveries was investigated by loading 10 mL of Milli-Q water spiked with 500 ng of BPA into the PPMIP-SPE cartridge, followed by rinsing with different volumes (1, 2, 3, and 4 mL) of ACN. The obtained BPA recoveries are given in Figure 3. Without any washing step, the BPA recovery was close to 100%, and no obvious reduction of BPA recoveries was observed when 1 and 2 mL of ACN were used. However, an obvious decrease of the BPA recoveries was observed when 3 and 4 mL of ACN were used. At last, 2 mL was selected as the proper volume of washing solvent.

#### 3.4.3. Selection of Elution Solvent Volume

The mixture solvent of methanol/TFA (98:2, *v*/*v*) was selected as the elution solvent. In order to optimize the elution solvent volume, 10 mL of Milli-Q water fortified with 500 ng of BPA were loaded onto the PPMIP cartridge, and different elution volumes (2, 3, 4, and 5 mL) of methanol/TFA (98:2, *v*/*v*) were tested. It was found that a volume of 4 mL was sufficient to elute the adsorbed BPA completely from the PPMIP cartridge. Consequently, 4 mL was selected as the elution solvent volume.

### 3.5. Analysis of Spiked Biological, Environmental and Food Samples

Using the developed HPLC method proposed in Section 2.6.3, BPA can be determined in about 7 min. Eight BPA standards with different concentrations of 0.02–2 mg·mL^−1^ were tested to determine the linearity, and satisfactory result was obtained with a correlation coefficient of 0.9998.

**Figure 4:**
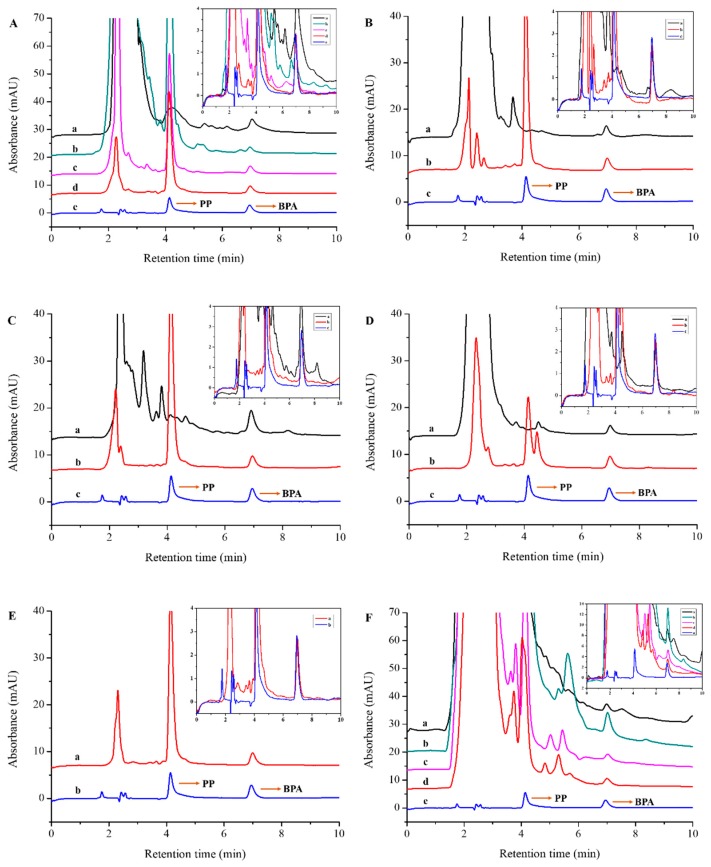
Chromatograms of the six investigated samples and standards. (**A**) Urine sample without PPMIP-SPE (a), urine samples after PPMIP-SPE at loading pHs of 3.0 (b), 6.0 (c), and 9.0 (d); (**B**) bovine serum sample without PPMIP-SPE (a), bovine serum sample after PPMIP-SPE (**b**); (**C**) sediment sample without PPMIP-SPE (a), sediment sample after PPMIP-SPE (b); (**D**) milk sample without PPMIP-SPE (a), milk sample after PPMIP-SPE (b); (**E**) edible oil sample after PPMIP-SPE (a); (**F**) soybean sauce sample without PPMIP-SPE (a), soybean sauce samples after PPMIP-SPE at loading pH of 3.0 (b), 6.0 (c), and 9.0 (d); (Ae, Bc, Cc, Dc, Eb, Fe) standard mixture of PP and BPA (at the bottom of each figure); insets: enlarged chromatograms displayed in an overlapping manner. All tested samples were spiked with 500 ng of BPA, and the concentrations of both PP and BPA in the standard mixture were 500 ng·mL^−1^.

To evaluate the applicability of the proposed method for complicated samples, the extraction of BPA in human urine (Figure 4A), bovine serum (Figure 4B), sediment (Figure 4C), milk (Figure 4D), edible oil (Figure 4E), and soybean sauce (Figure 4F) was performed. The insets in Figure 4A–E are enlarged chromatograms for different samples, which are used to discern the baseline noise. During the analysis procedure of biological samples, the existence of many interfering matrix components in human urine was demonstrated by the chromatogram of 1 mL urine dry residue spiked with 500 ng of BPA (Figure 4Aa and inset a). As can be seen from Figure 4Aa, especially Figure 4A inset a, the chromatographic peak of BPA was fully overlapped. At the stage of SPE optimization, a sample pH range of 3.0–9.0 was adopted. The chromatogram of spiked human urine sample after PPMIP-SPE at a loading pH of 3.0 showed poor clean-up efficiency (Figure 4Ab and inset b). When a loading pH of 6.0 was adopted, the clean-up efficiency was obviously improved (Figure 4Ac and inset c). In comparison with the chromatogram of the BPA standard (Figure 4Ae and inset e, 500 ng·mL^−1^), most of the interfering matrix components were eliminated when a sample loading pH of 9.0 was adopted (Figure 4Ad and inset d). This result could be attributed to the suppression of interaction between the ionized acidic interfering components of human urine under alkaline conditions with the pyridine groups of PPMIP [17,31]. At last, 9.0 was chosen as the loading pH of the urine sample.

Before PPMIP-SPE, the prepared bovine serum sample was concentrated to a 1 mL volume for HPLC analysis. As can be seen from the obtained chromatogram (Figure 4Ba and inset a), accurate quantification of BPA cannot be realized without using the SPE procedure, due to the interference of shoulder peak and baseline fluctuation around BPA. Comparing the chromatograms of the BPA standard (Figure 4Bc and inset c, 500 ng·mL^−1^) and the bovine serum sample treated with PPMIP-SPE (Figure 4Bb and inset b), the shoulder peak and baseline fluctuation around BPA was thoroughly eliminated, and no interference towards BPA quantification was observed after PPMIP-SPE.

The chromatogram of directly extracted sediments spiked with BPA is shown in Figure 4Ca and inset a, demonstrating the severe interference for BPA quantification as a result of the co-existing matrix components. In comparison, the chromatogram of sediment extract after PPMIP-SPE in Figure 4Cb and inset b shows good cleanup efficiency by comparison with the chromatogram of the BPA standard (Figure 4Cc and inset c, 500 ng·mL^−1^).

In comparison with the chromatogram of spiked BPA (500 ng·mL^−1^) in milk without SPE (Figure 4Da and inset a), the PPMIP-SPE procedure can be considered as an indispensable step for milk sample analysis because of the greatly improved recovery and clean-up effects (Figure 4Db and inset b). For the spiked edible oil sample, a one-step extraction of BPA using NaOH was performed before PPMIP-SPE. Under strong alkaline conditions, BPA could be completely ionized and exist as anionic forms, and thus could be transferred into the water phase. As can be seen from Figure 4Ea, especially Figure 4E inset a, no interference for the quantification of BPA in edible oil extract was observed after PPMIP-SPE. The pH effect on the clean-up effects for the soybean sauce sample was similar with that for human urine. As can be seen from the chromatogram of spiked soybean sauce without PPMIP-SPE (Figure 4Fa and inset a), severe baseline fluctuations were observed, and the chromatographic peak of BPA was completely overlapped. Poor clean-up efficiency was also obtained when a loading pH of 3.0 was adopted (Figure 4Fb and inset b). The clean-up efficiency was improved to some extent at the loading pH of 6.0, but baseline fluctuations could still be observed (Figure 4Fc and inset c), and low BPA recovery was obtained. When a loading pH of 9.0 was adopted, the clean-up efficiency was greatly improved (Figure 4Fd and inset d). As can be seen from Figure 4Fd,e and related insets (Figure 4F inset d, e), the baseline fluctuation was obviously suppressed, and no obvious interference towards BPA quantification was observed after PPMIP-SPE. It is worth noting that severe template leakage during the PPMIP-SPE procedure was observed for all chromatograms (see Figure 4A–E) by comparing them with the chromatograms of the PP standard (Figure 4Ae,Bc,Cc,Dc,Eb,Fe, 500 ng·mL^−1^). This result further confirms the necessity of adopting a dummy template for the preparation of MIPs.

To demonstrate the reliability of the developed PPMIP-SPE procedure, the BPA recoveries were determined at two different spiking levels (100 and 500 ng) for human urine, bovine serum, sediment, milk, edible oil, and soybean sauce samples. As can be seen from Table 1, the types of tested samples in this work were evidently more varied than in other related works. Before analysis, these tested samples were verified to be free of BPA. As can be seen from Table 2, satisfactory BPA recoveries in the range from 82.1% to 106.9% with RSD values below 7.7% were obtained when the developed PPMIP-SPE procedure was applied to the sample analysis. The limit of detection (LOD) of BPA were calculated to be 1.3 ng·mL^−1^ for bovine serum and milk samples, 2.6 ng·mL^−1^ for human urine and edible oil samples, 5.2 ng·mL^−1^ for soybean sauce samples, and 1.3 ng·g^−1^ for sediment samples (Table 2). These results are also superior or comparable to those reported in the literature using other preparation techniques (70 ng·mL^−1^ for water samples [32]) or other SPE materials (3.05 ng·mL^−1^ for milk samples [33]). The obtained results above demonstrated that a reliable and practical analytical method based on PPMIP-SPE purification, coupled with HPLC-DAD detection for the rapid determination of BPA from human urine, bovine serum, sediment, milk, edible oil, and soybean sauce samples was successfully established.

## 4. Conclusions

In this work, a novel dummy-template molecularly imprinted polymer for BPA was prepared by bulk polymerization using non-toxic PP as the template. By evaluating the *IF* values and sorption characteristics, the prepared PPMIP showed good selectivity and specific adsorption capacity for BPA. Furthermore, the developed PPMIP-SPE purification procedure showed good selectivity and clean-up efficiency for BPA from human urine, bovine serum, sediment, milk, edible oil, and soybean sauce samples. Here, the use of non-toxic PP as the dummy template not only avoided interference from template leakage of target analytes, but also made our detection method more environmentally friendly. In summary, the developed method of PPMIP-SPE purification, coupled with the HPLC-DAD detection method was accurate, sensitive, and reliable for the rapid determination of BPA in the six complex real samples.

## Figures and Tables

**Table 1:** Comparison of the prepared phenolphthalein imprinted polymer (PPMIP) in this work with other MIPs for the solid-phase extraction (SPE) of BPA in various samples.

Template Molecule	*IF* Value for BPA	Sample Type	Reference
BPA	6.4	Human urine	[17]
BPAP	About 4.0	River sediment	[18]
BPS	8.7	Tap and river water	[28]
BPB	About 5.0	River water	[29]
BPAF	Not shown	Environmental water	[30]
PP	9.0	Human urine, bovine serum, sediment, milk, edible oil and soybean sauce	This work

**Table 2:** Limits of detection (LODs), average recoveries, and relative standard deviations (RSDs, *n* = 3) of BPA obtained after the PPMIP-SPE of human urine, bovine serum, sediment, milk, edible oil, and soybean sauce samples at two different spiking levels.

Sample Type	LOD (ng·mL^−1^)	Spiked Level ^a^ (ng)	Sample 1		Sample 2		Sample 3	
			Recovery (%)	RSD (%, *n* = 3)	Recovery (%)	RSD (%, *n* = 3)	Recovery (%)	RSD (%, *n* = 3)
Human urine	2.6	100	97.3	6.2	90.5	1.2	91.4	3.2
		500	94.7	3.7	103.8	2.6	98.2	4.4
Bovine serum	1.3	100	95.1	5.8	99.1	2.9	97.3	1.6
		500	93.3	1.2	97.5	5.8	99.2	3.1
Sediment	1.3 ^b^	100	103.4	2.2	104.7	4.3	97.9	1.3
		500	91.2	3.5	95.7	1.6	98.1	5.7
Milk	1.3	100	98.8	1.5	96.4	6.3	98.1	3.3
		500	100.6	4.1	102.8	5.0	99.0	2.7
Edible oil	2.6	100	96.1	2.9	94.6	4.8	97.2	6.0
		500	103.2	7.7	106.9	6.4	100.4	5.2
Soybean sauce	5.2	100	83.6	2.1	82.1	1.8	87.2	3.6
		500	87.9	1.7	90.2	1.6	89.0	2.9

(^a^) For 1 mL of human urine, 2 mL of bovine serum, 2 g of sediment, 2 mL of milk, 1 mL of edible oil, and 0.5 mL of soybean sauce. (^b^) The LOD unit is ng/g for sediment samples.

## Supplementary Materials

The following are available online at http://www.mdpi.com/2073-4360/10/10/1150/s1, Figure S1: Molecular structures of BPA, PP, BPB, BPAF, BPS, E2, DES, and NP; Figure S2: Nitrogen adsorption–desorption isotherms of PPMIP (**a**), BPAMIP (**b**), and NIP (**c**) (inset: pore size distribution plot); Figure S3: *IF* values of BPA, BPB, BPAF, BPS, E2, DES, and NP for the PPMIP and BPAMIP.
