# Supplementary material for: A Phenolphthalein-Dummy Template Molecularly Imprinted Polymer for Highly Selective Extraction and Clean-Up of Bisphenol A in Complex Biological, Environmental and Food Samples
Source: Polymers (Basel). 2018 Oct 15;10(10):1150. doi: 10.3390/polym10101150 (PMC6403629; doi:10.3390/polym10101150)
Supplement: Supplementary file 1 [file polymers-10-01150-s001.pdf]

Article

Phenolphthalein-Dummy Template Molecularly Imprinted Polymer for Highly Selective Extraction and Clean-Up of Bisphenol A in Complex Biological, Environmental and Food Samples

Jiajia Yang [1,†], Yun Li [2,†], Chaonan Huang [2,3], Yanna Jiao [4] and Jiping Chen [2,*]

1 College of Materials Science and Engineering, Hebei University of Engineering, 199 South Guangming Street, Handan 056038, China; yjj177@163.com (J.Y.)
2 CAS Key Laboratory of Separation Sciences for Analytical Chemistry, Dalian Institute of Chemical Physics, Chinese Academy of Sciences, 457 Zhongshan Road, Dalian 116023, China; liyun@dicp.ac.cn (Y.L.); huangchaonan@dicp.ac.cn (C.H.)
3 University of Chinese Academy of Sciences, Beijing 100049, China
4 Inspection and Quarantine Technology Centre, Hunan Entry-Exit Inspection and Quarantine Bureau, Changsha 410004, China; 103993770@qq.com (Y.J.)
* Correspondence: chenjp@dicp.ac.cn (J.C.); Tel.: +86-411-8437-9562
† These authors contributed equally to this work

Supporting Information

Figure S1.

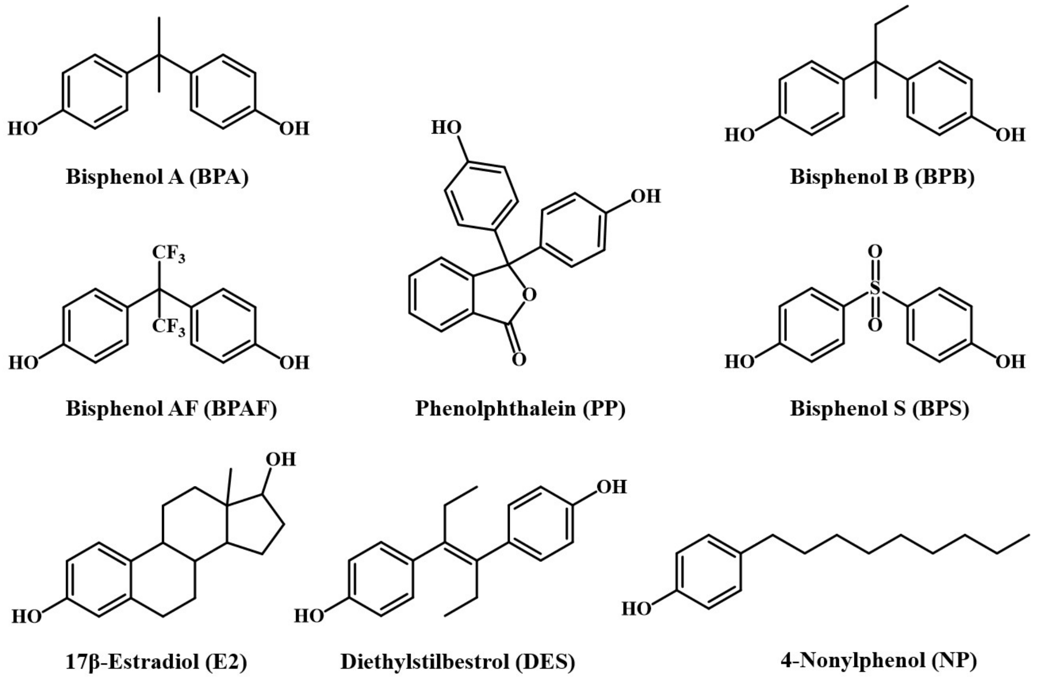

Figure S1. Molecular structures of BPA, PP, BPB, BPAF, BPS, E2, DES and NP.

Figure S2.

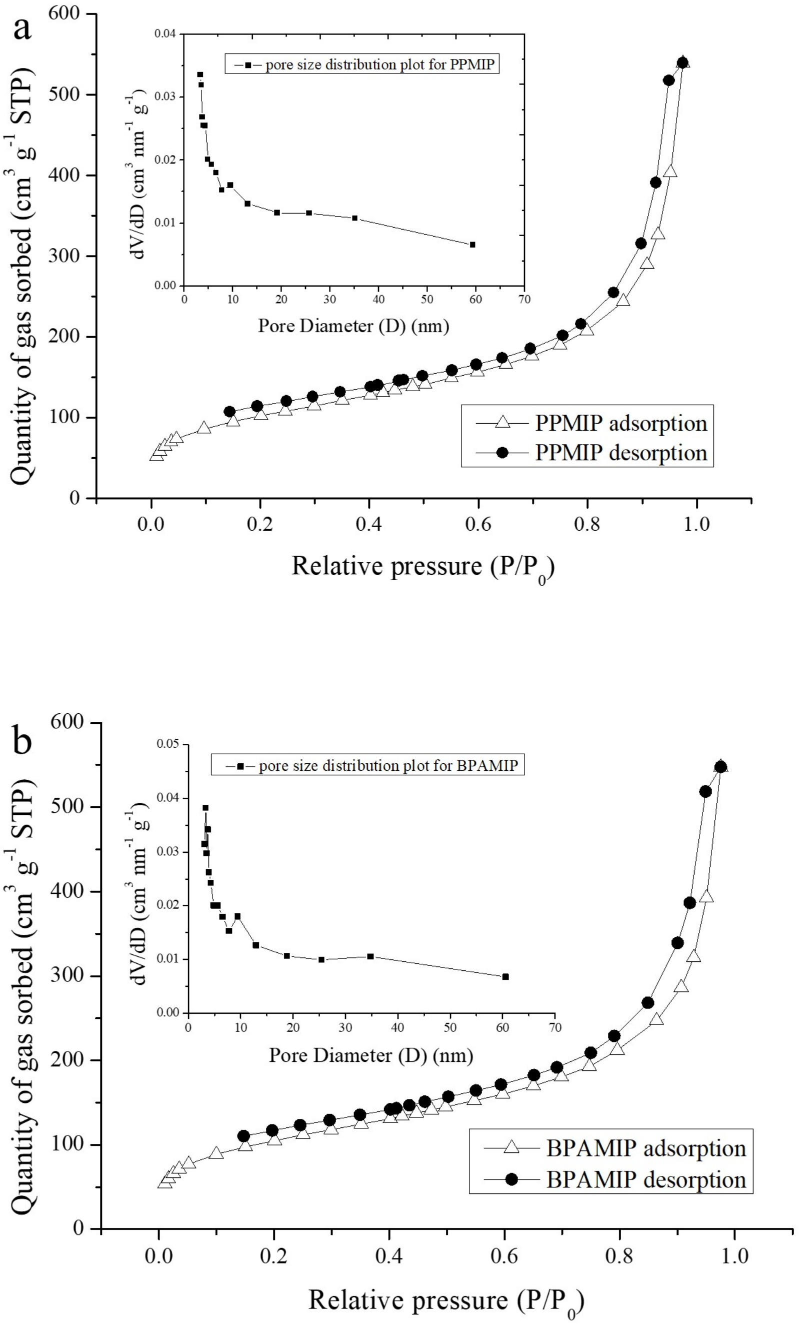
a
Quantity of gas sorbed (cm^3 g^{-1} STP)
Relative pressure (P/P$_0$)
PPMIP adsorption
PPMIP desorption
pore size distribution plot for PPMIP
dV/dD (cm^3 nm^{-1} g^{-1})
Pore Diameter (D) (nm)
b
Quantity of gas sorbed (cm^3 g^{-1} STP)
Relative pressure (P/P$_0$)
BPAMIP adsorption
BPAMIP desorption
pore size distribution plot for BPAMIP
dV/dD (cm^3 nm^{-1} g^{-1})
Pore Diameter (D) (nm)

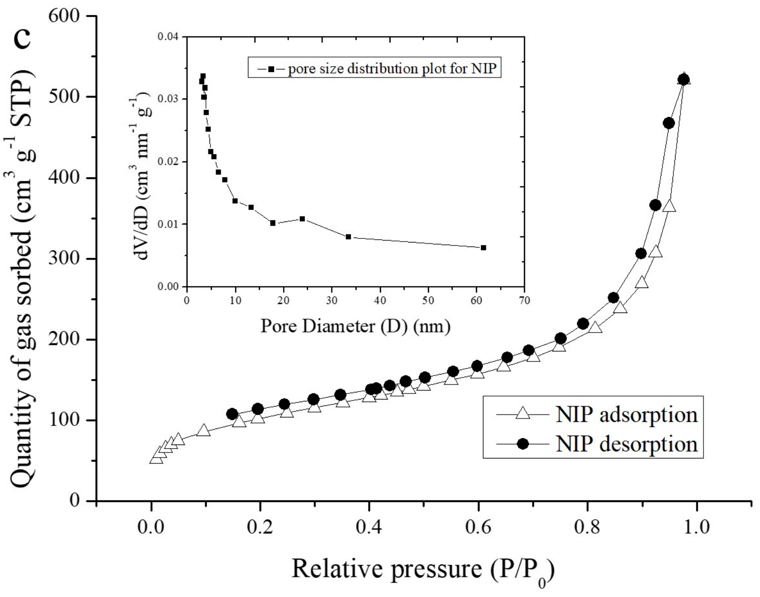

Figure S2. Nitrogen adsorption-desorption isotherms of PPMIP (a), BPAMIP (b) and NIP (c) (inset: pore size distribution plot).

Figure S3.

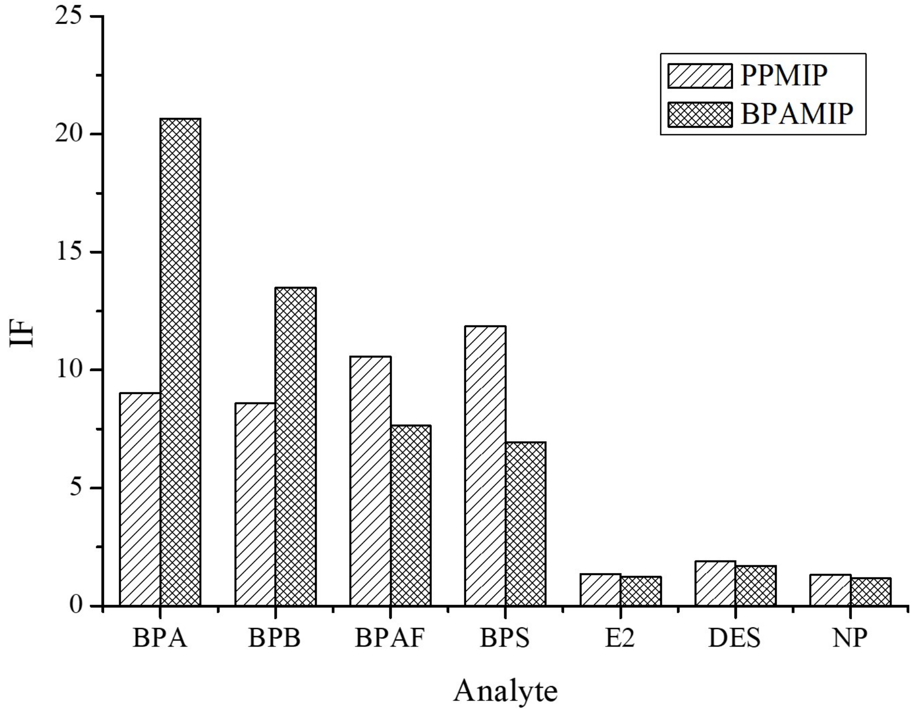

Figure S3. IF values of BPA, BPB, BPAF, BPS, E2, DES and NP for the PPMIP and BPAMIP.
